# Delayed total hip arthroplasty after failed treatment of acetabular fractures: an 8- to 17-year follow-up study

**DOI:** 10.1186/s13018-018-0909-8

**Published:** 2018-08-22

**Authors:** Tao Wang, Jun-Ying Sun, Jun-Jun Zha, Chao Wang, Xi-Jiang Zhao

**Affiliations:** 1 Department of Orthopedic Surgery, Affiliated Hospital of Jiangnan University, 200 Huihe Rd, Wuxi, 214062 Jiangsu China; 2 Department of Orthopedic Surgery, The First Affiliated Hospital of Soochow University, 188 Shizi Street, Suzhou, 215006 Jiangsu China

**Keywords:** Acetabular fracture, Total hip arthroplasty, Cementless cup, Ceramic-on-ceramic

## Abstract

**Background:**

Delayed total hip arthroplasty (THA) is a reliable procedure following failed treatment of acetabular fractures. The aim of the present study was to evaluate the influence of the type of fracture treatment and modern ceramic bearing on the clinical outcomes of delayed THA.

**Methods:**

Between January 1997 and January 2008, 33 patients (33 hips) underwent cementless THA after failed acetabular fractures. Twenty-one were initially treated by open reduction internal fixation (ORIF) and 12 had non-ORIF. Joint articulation was either conventional metal-on-polyethylene (MOP) or ceramic-on-ceramic (COC). Intraoperative measures and preoperative and follow-up clinical, radiological, and functional outcomes were compared between the ORIF and non-ORIF groups.

**Results:**

Surgery duration, blood loss, and transfusion requirement were greater in the ORIF group than in the non-ORIF group (*p* < 0.05). Significant improvement in Harris Hip Scores was seen post-surgery in both groups. However, a significant difference in the mean Harris Hip Score was not observed between the two groups (*p* = 0.57). Six patients in the ORIF group required acetabular reconstructive procedures to address bony defects compared to seven patients in the non-ORIF group (*p* = 0.09). The rate of anatomical restoration was 58.3% (7/12) in the non-ORIF group and 42.9% (9/21) in the ORIF group (*p* = 0.12). Radiolucent lines were observed in the MOP group and none in the COC group. Overall survival rate was similar in both groups (*p* = 0.85): 89.3% in the ORIF group and 87.5% in the non-ORIF group.

**Conclusion:**

Delayed THA with previous acetabular fractures is a challenging procedure. Initial fracture treatment does not influence the outcome of delayed THA, and modern ceramic bearing has promising results in the long-term follow-up.

## Background

Acetabular fractures are serious injuries which can lead to progressive impairment of hip function [1]. Anatomic reduction with rigid internal fixation of the acetabulum has been shown to restore hip function and prevent long-term complications. Unfortunately, many patients with fractures of the acetabulum still suffer posttraumatic arthritis or femoral head necrosis regardless of whether operative or non-operative intervention was chosen as the initial treatment. These irreparable complications may occur as a result of residual articular incongruity, early articular cartilage damage, inaccurate placement of implant fixation, and disruption of femoral head blood provision. Even when near-anatomic reductions are achieved, the reported incidence of posttraumatic arthritis has been varied between 27 and 37% [2], with the incidence of subsequent total hip arthroplasty (THA) ranging from 8 to 23% [2–4].

When posttraumatic arthritis and femoral head necrosis occur, THA is a rational salvage procedure. Retained internal fixation implants, scar tissue, and residual acetabulum bone defects cause subsequent THAs to be more complex than routine THA [5, 6]. Moreover, the results of these THAs presented to be inferior to THA performed after primary osteoarthritis [7–9], attributable to the extent of loss of bone stock and abnormal anatomy after trauma [9–11]. Therefore, some authors recommended that initial open reduction internal fixation (ORIF) is essential to restore the bony anatomy for subsequent THA, decreasing its complexity and improving component survival [12–14]. Conversely, others have postulated that initial ORIF increases the rate of infection, blood loss, and surgery time [6, 15]. Furthermore, there is no explicit evidence that ORIF improves the success rate of subsequent THA [16].

Additionally, patients with acetabular fractures are generally young and have posttraumatic osteoarthritis that results from a high activity level. A series reports high failure rates of subsequent THA, which have been attributed to younger age and increased activity [9, 11]. Therefore, in those patients, alternative bearing surfaces should reduce polyethylene wear and osteolysis that have been present in previous studies using traditional metal-on-polyethylene (MOP). Recently, modern ceramic-on-ceramic (COC) THA has demonstrated decreased risk of wear-induced osteolysis over MOP and then improved the long-term outcomes of THA [17, 18].

Therefore, the primary aim of the present study was to evaluate the influence of the mode of treatment of fracture, conservative treatment or ORIF, on the clinical outcomes of salvage THA. The secondary aim was to evaluate the long-term results associated with modern COC THA after failed acetabular fractures.

## Methods

All patients who underwent THA for posttraumatic osteoarthritis or femoral head necrosis due to acetabular fractures during the period from January 1997 to January 2008 were eligible for inclusion in this study. Exclusion criteria were incomplete radiographic or clinical date and without adequate follow-up. Two patients (2 hips) had died of causes unrelated to surgery. Five patients (5 hips) were lost to follow-up. Thus, 33 remaining patients (33 hips) were available for review. Patient demographics and characteristics of the fractures are summarized in Table 1. This study was approved by our institutional review board.

**Table 1:** Patient demographics and fracture features

Gender	
Male/female	21/12
Mean age (years)	45.1 ± 9.3 (range, 25–68)
Mean body mass index (kg/m^2^)	23.5 ± 2.9 (range, 17.0–32.9)
Interval time between fracture and THA (month)	58 (range, 4–240)
Preoperative diagnosis	
Posttraumatic osteoarthritis	23
Femoral head necrosis	10
Fracture patterns	
Simple factures	
Posterior column	4
Posterior wall	7
Anterior column	2
Anterior wall	0
Transverse	3
Complex fractures	
Posterior wall + posterior column	3
Transverse + posterior wall	6
Both column	4
T-shaped	2
Anterior column + posterior hemitransverse	3
Fracture treatment	
ORIF	21
Non-ORIF	12

### Surgical technique and implants

Delayed THA was performed through a posterior approach in 25 hips, and a modified Hardinge approach in 8 hips. The acetabulum was prepared for routine primary THA. Retained hardware or heterotopic bone was not routinely removed unless it interfered with reaming and placement of the cup. Acetabular bone deficiency was classified based on American Academy of Orthopedic Surgeons (AAOS) classifications [19]. We adopted impaction bone grafting (IBG) combined with a cementless cup to address acetabular bone defects with segments of bone or morselized cancellous bone taken from the resected femoral head. Cementless acetabular fixation was used in all cases using a press-fit technique. If the initial press-fit fixation did not provide sufficient cup stability, additional screw fixation was conducted. Joint articulation was either MOP or COC. MOP was used in 12 hips. A Synergy stem and Reflection acetabular cup (Smith & Nephew, USA) were used in six hips, a Self-Locking stem and SPH cup (Lima, Italy) was used in four hips, and a Bi-Metric stem and Universal cup (Biomet, USA) in two hips. COC was used in 21 hips, in which a C2 stem and “Sandwich” cup (Lima, Italy) was used in five hips, an F2L stem and “Sandwich” cup (Lima, Italy) in four hips, and an S-ROM stem and Duraloc cup (Depuy, USA) in 12 hips.

### Perioperative regimen

Patients received antibiotic prophylaxis at 0.5 h before surgery and within the first 24 h postoperatively. Low-molecular-weight heparin was routinely administered for preventing thromboembolism. No patient received prophylaxis against heterotopic ossification. All patients were allowed to do quadriceps strengthening exercises and passive movements following surgery. Patients that had received a bone graft were instructed to restrict movement to touch-down weight-bearing for 6 weeks then gradually increase this to full weight-bearing thereafter.

### Clinical and radiographic evaluation

**Fig. 1:**
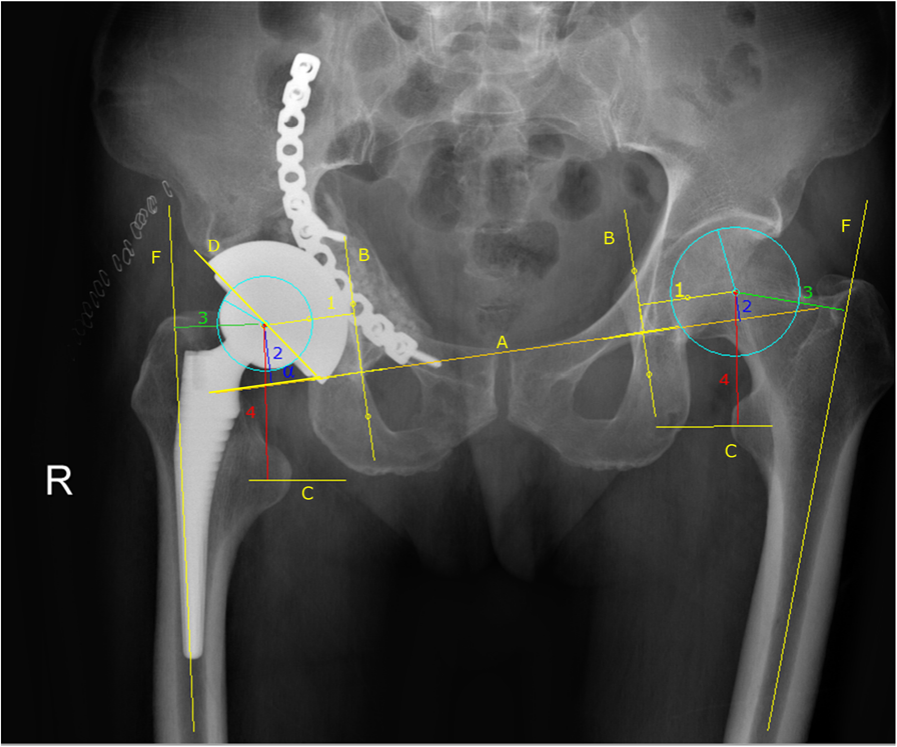
Postoperative radiograph after THA with impacting bone grafting combined with a cementless cup. A, horizontal teardrop line; B, vertical teardrop line; C, midline lesser trochanter; D, a line along the lateral surface of the acetabular component; F, femoral shaft line. 1, horizontal hip center of rotation; 2, vertical hip center of rotation; 3, horizontal femoral offset; 4 vertical femoral offset; α, the angle between a line drawn between line A and line D

Clinical and radiographic examinations were performed at 6 weeks, 6 months, 1 year, and annually thereafter. Hip function was assessed using the Harris Hip Score (HHS) [20]. All plain radiographs qualified for evaluation of biomechanical reconstruction are shown in Fig. 1. Linear polyethylene wear was measured using the method of Pollock et al. [21] using Digimizer software. Radiolucent lines and osteolysis were evaluated on postoperative serial radiographs according to the method of DeLee and Charnley [22] and Gruen et al. [23] for the acetabulum and femur, respectively. Bony ingrowth was described according to the criteria of Engh et al. [24]. Heterotopic ossification was classified according to the system of Brooker et al. [25].

### Statistical methods

Statistical analyses were performed using SPSS v19.0 (SPSS, USA) statistical software. All data were expressed as mean ± standard deviation (SD) and were tested for equal variance using the Levene test. Categorical variables were evaluated using a chi-square test or Fisher’s exact test for statistical significance. Continuous variables were assessed using a Student *t* test. The level of significance was set at *p* < 0.05. Survivorship analysis of reconstructions was assessed by the Kaplan–Meier. The endpoint was defined as a revision for any reason or definite radiographic signs of loosening.

## Results

### Clinical outcomes

Mean follow-up time was 11.5 ± 3.0 years (range, 8–17 years) after primary THA. The mean duration between initial fracture and requirement for subsequent arthroplasty was not significantly different between the ORIF group and the non-ORIF group (52.8 months vs 62.4 months). Union was achieved in all fractures in the ORIF group, but four (33.3%) non-unions were in the non-ORIF group. For subsequent THA, the non-union fractures were treated with rigid internal fixation and bone grafting. In the ORIF group, the retained hardware was removed either in part or in its entirety in four patients (4/21) who had undergone prior ORIF of acetabular fractures. Six patients (6/21) in the ORIF group required acetabular reconstruction to address bony defects compared to seven patients (7/12) in the non-ORIF group, although the difference was not statistically significant (*p* = 0.09). The comparison revealed that longer surgery occurred in the ORIF group (189 min for ORIF vs 143 min for non-ORIF, *p* = 0.02) and greater blood loss (1289 ml for ORIF vs 750 ml for non-ORIF, *p* < 0.01). Differences in the average perioperative transfusion requirements were also found (6.6 units for ORIF vs 3.5 units for non-ORIF, *p* < 0.01) (Table 2).

**Table 2:** Perioperative data for ORIF and non-ORIF groups

Variable	ORIF group	Non-ORIF group	*p* value
Sex (M/F)	13/8	8/4	0.78
Age at THA (years)	44.9 ± 10.5	45.5 ± 7.2	0.86
Interval time between fracture and THA (months)	52.8 ± 12	62.4 ± 20	0.09
Bone defects	6	7	0.09
Fracture pattern			
Simple/complex	8/13	7/5	0.26
Harris hip score			
Preoperative	45.9 ± 12.1	40.8 ± 11.3	0.24
At last follow-up	89.0 ± 5.4	87.9 ± 4.8	0.57
Surgery duration (min)	189 ± 57	143 ± 32	0.02
Blood loss (ml)	1289 ± 429	750 ± 145	0.00
Perioperative transfusion requirements (u)	6.6	3.5	0.00

*M* male, *F* female

Mean ± SD

Mean HHS improved across all patients from 44.0 ± 11.9 points (range, 27–58 points) preoperatively to 88.6 ± 5.1 points (range, 74–94 points) at the final follow-up. Excellent scores were observed in 24 hips, good in 7, and fair in 2. In the ORIF group, mean HHS increased from 45.9 ± 12.1 points (range, 27–58 points) to 89.0 ± 5.4 points (range, 74–94 points) (*p* < 0.05). In the non-ORIF patients, it improved from 40.8 ± 11.3 points (range, 29–58 points) to 87.9 ± 4.8 points (range, 79–94 points) (*p* < 0.05). However, difference in the mean HHS at final follow-up was not observed between the two groups (*p* = 0.57) (Table 2).

### Radiographic results

**Fig. 2:**
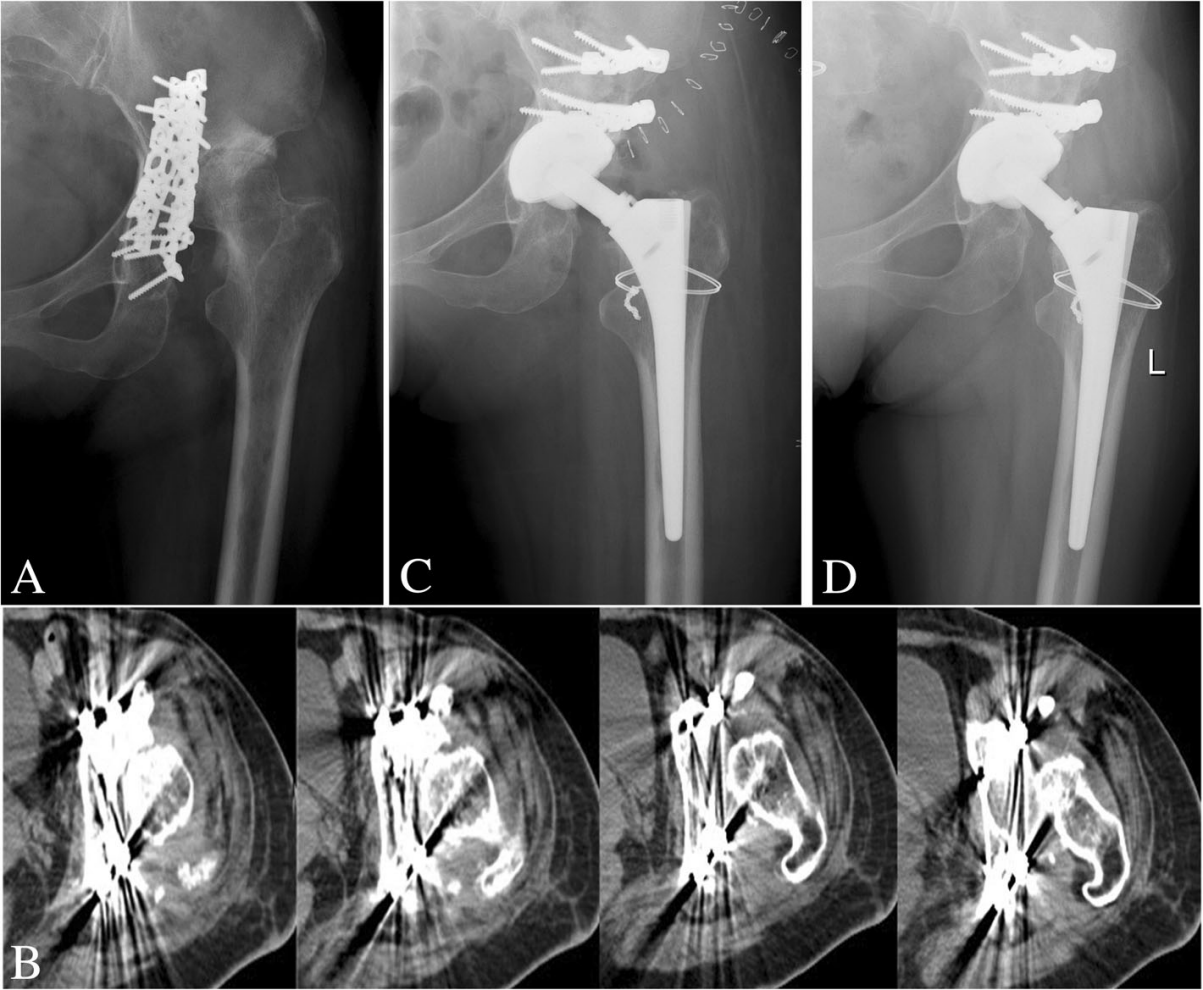
Delayed total hip arthroplasty (THA) in a 51-year-old woman with posttraumatic arthritis secondary to acetabular fracture. **a** Preoperative radiograph. **b** Computed tomographic scans of the hip revealed the posterior acetabular wall defect. **c** Immediate postoperative X-ray after acetabular reconstruction with the structure bone graft combined with cementless cup. **d** A 15 years’ follow-up X-ray showed the graft had completely united and no loosening or osteolysis around the acetabular or femoral component

**Fig. 3:**
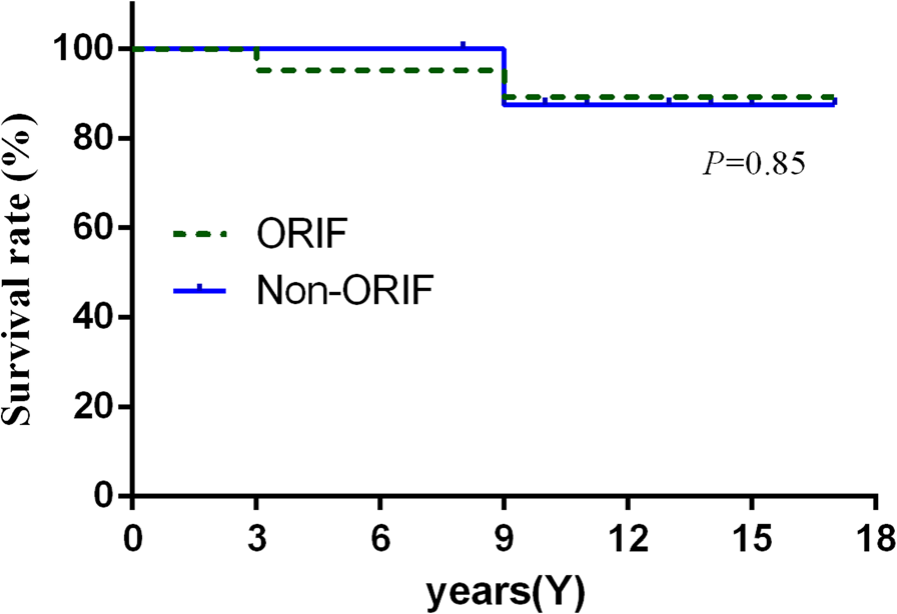
Kaplan–Meier survival curves at a mean 11.5 years for all patients who performed THA after acetabular fracture with implant revision for any reason as an endpoint and due to loosening at the time follow-up as the other endpoint, respectively

Mean abduction angle was 33.8° ± 8.5° (range, 16–54°). At the final radiographic follow-up, surviving implants showed radiographic evidence of stable bony ingrowth (Fig. 2). All bone grafts united with the host bone, without any graft resorption observed. No acetabular component loosening was observed in either group. In the ORIF group, two acetabular revisions were performed due to infection and a ceramic liner fracture. In the non-ORIF group, one patient had ceramic liner fracture and underwent acetabular revision. Failure defined as revision of the acetabular component for any reason, the Kaplan–Meier survivorship of the prostheses was 88.9% (95% CI 76.9~100%). Moreover, overall survival rate was similar in both groups (*p* = 0.85): 89.3% in the ORIF group and 87.5% in the non-ORIF group (Fig. 3).

The impact of THA on biomechanical reconstruction parameters observed in the contralateral hip is shown in Table 3, although there were no significant differences between the non-ORIF and ORIF groups. Among the 33 patients, reconstructed hip center within 20 mm of vertical and horizontal symmetry happened in 27 patients compared with the contralateral hip, including 16 patients (7 non-ORIF vs 9 ORIF) with anatomical restoration and 11 patients (5 non-ORIF vs 6 ORIF) with a reconstructed hip center within 10–20 mm of vertical and horizontal symmetry. The remaining six patients, who were all in the ORIF group, had a reconstructed hip center more than 20 mm beyond vertical or horizontal symmetry, or both. Therefore, the rate of anatomical restoration was 58.3% (7/12) in the non-ORIF group and 42.9% (9/21) in the ORIF group (*p* = 0.12). Anatomical restoration was not related to the method of fracture treatment (*r* = 0.248, *p* = 0.163).

**Table 3:** Biomechanical parameters on the operated hip compared to the native contralateral hip (unilateral hip arthroplasty). Data are presented as mean (SD, range)

Parameter	Non-ORIF (*n* = 12)	ORIF (*n* = 21)	*p* value
Horizontal hip center of rotation	+ 2.6 (± 0.59, − 7.9~10.3)	− 1.9 (± 8.3, − 20.6~12.2)	0.130
Vertical hip center of rotation	+ 5.2 (± 7.9, − 11.3~14.2)	+ 10.7(± 11.0, − 21.7~27.2)	0.134
Horizontal femoral offset	+ 1.4 (± 8.6, − 12.9~16.6)	+ 4.2(± 10.3, − 25.5~17.7)	0.29
Vertical femoral offset	+ 5.0 (± 12.9, − 19.4~24.2)	+ 9.1(± 7.8, − 13.2~25.0)	0.48

**Fig. 4:**
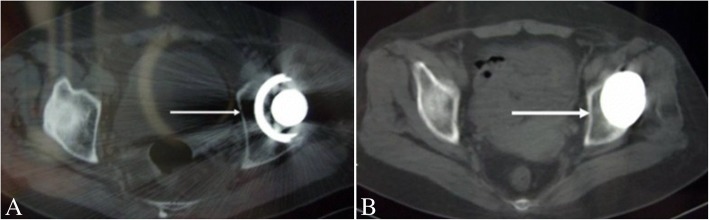
**a** Local osteolysis around the acetabular was observed by computed tomography (CT) in the metal-on-polyethylene group. **b** No evidence of osteolysis around acetabular component was observed in the ceramic-on-ceramic group (white arrow)

Average rate of conventional linear polyethylene wear was 0.22 ± 0.05 mm/year with a mean of 11.5 years in the MOP group. At the final follow-up, linear wear of the polyethylene insert was measured > 0.2 mm/year in eight cases. Among them, six acetabular components formed partial radiolucent lines at the bone-implant interface, observed in zone I in four hips and in zone III in two hips. On the femoral side, two stems had radiolucent lines in Gruen zone 1. This had not affected acetabular or femoral component stability, and they had not been revised. In contrast, no radiolucent lines or osteolysis was observed in any patient in the COC group (Fig. 4).

### Complications

Heterotopic ossification was observed in ten patients (seven in the ORIF group, three in the non-ORIF group). No heterotopic bone caused any adverse clinical effects. One posterior dislocation occurred in the ORIF group, which was successfully managed with closed reduction. The sciatic nerve was injured before THA in two patients in the ORIF group. No patient had new or progressive sciatic nerve symptoms after THA. Additionally, three revisions were performed (two ORIF group and one non-ORIF). One patient in the ORIF group underwent a successful two-stage revision hip arthroplasty to control infection. Two “sandwich” liner fractures occurred in both ORIF and non-ORIF groups.

## Discussion

When posttraumatic osteoarthritis develops secondary to acetabular fracture, THA is considered a reliable procedure to relieve pain and restore function [8, 26, 27]. Failed acetabular fractures after non-operative treatment often have bone defects, suffer non-union, or have residual pelvic deformity from malunion [11]. Similarly, following initial ORIF treatment, THA procedures not only faced the same difficulty as non-operatively treated fractures, but they also suffered problems related to previous surgery such as proliferative scar tissue, heterotopic bone formation, or obstructive hardware [6, 8, 9, 28]. In our study, we observed that the surgery duration and blood loss were greater in the ORIF group than in the non-ORIF group. However, no significant difference in average HHS was observed at the final follow-up between the two groups (ORIF group 89 vs non-ORIF 87.9, *p* = 0.57). In brief, performing a salvage THA following acetabular fracture is a challenging procedure. ORIF prior to THA resulted in more complex surgery which did not improve the final clinical outcome.

Acetabular bone defects and abnormal anatomy are contributing factors to the inferior THA outcomes experienced in the setting of previous acetabular fractures [7, 27]. Weber et al. [8] also demonstrated that large deficiencies in acetabular bone were associated with a poorer rate of long-term outcome after delayed THA. To restore bone stock and minimize acetabular deformity, various reconstruction techniques have been described to treat this issue effectively. IBG, a widely accepted technique for acetabular reconstruction after bone stock loss in revision THA, represents one option. Postoperative longevity of the acetabular component achieved from this technically demanding procedure is well documented in previous literature [29]. Based on this favorable experience, we reconstructed the acetabulum using the IBG technique with a cementless hemispherical cup in this study, obtaining sufficient host bone coverage and stability fixation (Fig. 2). Additionally, our results of THA in patients who were treated with ORIF for acetabular fractures demonstrated that ORIF did not reduce the incidence of bone defects compared with non-ORIF (6/21 vs 7/12, *p* = 0.09). Furthermore, the incidence of anatomical restoration was 58.3% (7/12) in the non-ORIF group and 42.9% (9/21) in the ORIF group (*p* = 0.12). It was shown that anatomical restoration was not associated with fracture treatment (*r* = 0.248, *p* = 0.163). What is more, there was also no clear evidence that ORIF could improve the long-term outcomes of the subsequent THA. Ranawat et al. [16] also hypothesized that initial ORIF was not an essential procedure before THA and that anatomical restoration was not related to fracture treatment. Moreover, there was an increased risk of infection, peri-articular ossification, and scar tissue, and the presence of retained hardware increased the duration of surgery and blood loss [6, 28].

Ranawat et al. [16] reported that acetabular component survival rate was 97% with aseptic loosening and 79% with revision for any reason. Bellabarba et al. [13] also described a 10-year survival rate of 97% after uncemented acetabular reconstruction. In our study, we demonstrated a cementless acetabular component survival rate of 100% with loosening and 88.9% with revision for any reason, because of two “sandwich” ceramic liner fractures due to part design defects [30, 31]. After performing revision surgery, those two hips had well-fixed implants with no radiolucencies and had good clinical results in the follow-up study. Furthermore, acetabular component survival in both groups was similar regarding any reason at a mean follow-up of 11.5 years (Fig. 3). In brief, complex reconstruction in the non-ORIF group did not affect component survival and none of the hips were revised because of aseptic loosening.

Wear debris from conventional MOP can cause extensive osteolysis, threatening the long-term survival of cementless cups, especially in young patients [32]. Berry and Halasy [33] reported that 67% of revisions were associated with polyethylene wear and osteolysis. Roth et al. [26] reported that survivorship of the acetabular component declined from 87% at 10 years to 57% at 20 years for polyethylene wear or loosening. Similarly, our results revealed that 8 of 12 patients who underwent THA with conventional MOP had partial radiolucent lines at the bone-implant interface (Fig. 4). The most likely explanation for periprosthetic osteolysis in these patients is low patient age (average 45 years) combined with high activity level. Conversely, we did not observe osteolysis in any patient in the ceramic-on-ceramic group. Our previous study demonstrated that THAs using COC suffer significantly reduced wear and demonstrated improved component longevity [17]. On the basis of the data presented, therefore, one could speculate that optimization of long-term results with COC surfaces has the potential to solve the most common problems that result in revision surgery being required in these patients (aseptic loosening and osteolysis).

Our study may have some limitations. First, this was a retrospective study and not a prospective randomized study, which increased the possibility of selection bias. Secondly, the number of patients in the study was relatively small, and further studies involving more participants are anticipated. Finally, the acetabular components used in this study were not uniform and thus may compromise the robustness of the results.

## Conclusion

In conclusion, Delayed THA with previous acetabular fractures is a challenging procedure. Our study found the initial fracture treatment does not influence the functional results and component survival of subsequent THA at long-term follow-up. Although acetabular osteolysis was observed in our patients, we believe that cementless THA with modern ceramic bearing surfaces will eliminate periprosthetic osteolysis and further improve long-term results.

## Abbreviations

AAOS: American Academy of Orthopedic Surgeons
COC: Ceramic-on-ceramic
IBG: Impaction bone grafting
MOP: Metal-on-polyethylene
ORIF: Open reduction internal fixation
THA: Total hip arthroplasty
